# Immediate Renal Denervation After Acute Myocardial Infarction Mitigates the Progression of Heart Failure via the Modulation of IL-33/ST2 Signaling

**DOI:** 10.3389/fcvm.2021.746934

**Published:** 2021-10-01

**Authors:** Han Chen, Rui Wang, Quan Li, Jiasheng Yin, Zhenyi Ge, Fei Xu, Tongtong Zang, Zhiqiang Pei, Chaofu Li, Li Shen, Junbo Ge

**Affiliations:** ^1^Department of Cardiology, Zhongshan Hospital, Fudan University, Research Unit of Cardiovascular Techniques and Devices, Chinese Academy of Medical Sciences, Shanghai, China; ^2^National Clinical Research Center for Interventional Medicine, Shanghai, China; ^3^Shanghai Institute of Cardiovascular Diseases, Shanghai, China; ^4^Department of Echocardiography, Zhongshan Hospital, Fudan University, Shanghai, China

**Keywords:** renal denervation, acute myocardial infarction, heart failure, myocardial remodeling, fibrosis, soluble ST2 (sST2)

## Abstract

**Objective:** Previous studies have demonstrated the protective effects of renal denervation (RDN) in pre-existing heart failure, but the effects of immediate RDN after acute myocardial infarction (AMI) on subsequent cardiac remodeling have not been reported. This study aimed to investigate the cardioprotective effects of immediate RDN after AMI and its underlying mechanism.

**Methods:** AMI was induced by intracoronary gelatin sponge embolization in 14 Shanghai white pigs that were randomized to undergo either renal angiography (AMI+sham group) or RDN (AMI+RDN group) after 1 h of hemodynamic monitoring. Cardiac function of the two groups was measured at baseline, 1 h post-AMI and at the 1 month follow-up (1M-FU) by transthoracic echocardiography (TTE). Plasma NT-proBNP, soluble ST2 (sST2), norepinephrine (NE), and renin-angiotensin-aldosterone system activity were detected simultaneously. The renal cortex was harvested for NE measurement after the 1M-FU, and the renal arteries were stained with tyrosine hydroxylase for the evaluation of sympathetic activity. Heart tissues in the non-ischemic areas were collected to assess histological and molecular left ventricular (LV) remodeling by pathological staining, RT-PCR, and western blotting.

**Results:** There was no difference in the hemodynamic stability or cardiac function between the two groups at baseline and 1 h post-AMI. Six pigs from each of the two groups completed the 1M-FU. TTE analysis revealed the improved cardiac function of immediate RDN in the AMI+RDN group and circulating NT-proBNP levels were lower than those in the AMI+sham group. Further analysis showed significantly less interstitial fibrosis in the remote non-ischemic myocardium after immediate RDN, together with decreased cardiomyocyte hypertrophy and inflammatory cell infiltration. sST2 levels in circulating and myocardial tissues of animals in the AMI+RDN group were significantly higher than those in the AMI+sham group, accompanied by corresponding alterations in IL-33/ST2 and downstream signaling.

**Conclusions:** Immediate RDN can improve cardiac function and myocardial remodeling after AMI via modulation of IL-33/ST2 and downstream signaling.

## Introduction

Although percutaneous coronary intervention (PCI) have significantly reduced acute mortality rates of acute myocardial infarction (AMI) by timely revascularization and myocardial salvage, ventricular remodeling afterwards, which involves cardiomyocyte hypertrophy, chronic inflammatory response, and the progression of fibrosis, can cause deteriorating cardiac function and even death (1–5). Additionally, recent studies have reported that an overactivated neurohumoral system plays a deleterious role in ventricular remodeling and many targeted drugs have be developed (6–8). However, due to drug tolerance and compliance difficulties, heart failure (HF) after AMI is still poorly controlled in the population worldwide, which is a substantial concern for society and also a strain from economic and healthcare perspectives (9, 10).

Denervation of sympathetic nerves around renal arteries (renal denervation; RDN) results in reduced activation of the sympathetic nervous system (SNS) and renin-angiotensin-aldosterone system (RAAS) (11). Studies on animal models have reported that RDN is beneficial in the treatment of diseases associated with SNS and RAAS hyperactivation in addition to its use for hypertension treatment (12–16). Among these effects, the cardioprotective effect of RDN on pre-existing HF has been confirmed (17, 18).

Inspired by the idea that concomitant RDN with pulmonary vein isolation (PVI) can further reduce the recurrence rate of atrial fibrillation, we brought up the hypothesis of a “one-stop” treatment for HF after AMI by performing RDN with a cryoablation catheter immediately after the onset of AMI, rather than for chronic ischemic HF (19). Therefore, in this study, we evaluated the cardioprotective effect of immediate RDN after AMI in a swine model and the possible mechanism on adverse myocardial remodeling.

## Materials and Methods

### Animals

A total of 14 Shanghai white pigs of either sex, with body weights ranging from 30 to 35 kg, were used in this study. All animals used in this study were cared for and handled according to the National Institutes of Health Guidelines. The study protocol was reviewed and approved by the Animal Care and Use Committee of Zhongshan Hospital, Fudan University.

### Study Design

An AMI was induced by occlusion of the proximal left anterior descending coronary artery (LAD) with gelatin sponges and set as the baseline. One hour after AMI, the surviving pigs were randomized into two groups that underwent either RDN with a cryoablation balloon catheter (CryoFocus, Shanghai, China) (AMI+RDN group) or renal angiography (AMI+sham group). All animals were sacrificed at 1 month follow-up (1M-FU), and the cardiac tissue was collected for further molecular or histological analysis. Transthoracic echocardiography (TTE) was performed at baseline, 1 h post-AMI and at the 1M-FU. Furthermore, peripheral blood samples were obtained at the same time points to assess cardiac injury and function.

### AMI Induction

The AMI was induced by intracoronary gelatin sponge embolization as described previously, and the total procedure lasted ~10 min (20). The animals were fasted for 12 h with unrestricted access to water. Anesthesia was induced by intramuscular injection of 6.0 mg/kg Zoletil® (a mixture of tiletamine hydrochloride and zolazepam hydrochloride, Virbac Laboratory, Carros, France) followed by an intravenous injection of propofol (5 mg/kg). A volatile anesthetic with isoflurane (1.5–2%) was used to maintain general anesthesia. An unfractured heparin bolus of 200 U/kg was administered after sheath placement in the right femoral artery. Invasive monitoring of aortic pressure was obtained using a pressure sensor (Transpac, ICUMEDICAL, CA, USA) and recorded by Mac-lab hemodynamic recording system (GE healthcare, MA, USA). A 6F SAL catheter (Medtronic, MN, USA) was introduced through the arterial sheath to the left main coronary artery. After angiography, a 1.8F Finecross microcatheter (Terumo, Tokyo, Japan) was placed in the middle of the LAD (between the first and second diagonal branches) under the guidance of a 0.014” guidewire. A mixture of gelatin sponge particles (300–1,000 μm) and heparinized contrast media was slowly injected through the microcatheter under fluoroscopy. The injection was stopped after total occlusion of the middle LAD. The pigs were carefully monitored under volatile anesthesia for 60 min, and then randomized for further operation. After the 1M-FU, the pigs were sacrificed by rapid intravenous injection of 20 mL of 10 % potassium chloride, and samples were harvested for analysis.

### Renal Denervation

RDN was performed using a cryoablation balloon catheter as previously described (21, 22). After randomization, pigs in the AMI+sham group underwent renal angiography with a 6F JR 4 guiding catheter (Medtronic). For pigs in the AMI+RDN group, a 7F sizable cryoablation balloon catheter was selected based on the diameter of the middle to distal part of the main renal arteries and was introduced into the renal artery via the same percutaneous femoral access as in the AMI modeling. After confirming a complete attachment to the artery wall, the balloon was inflated with liquid nitrogen, and a complete RDN for one side of the renal artery took ~5 min. Additional renal angiography was performed before catheter removal to confirm the absence of complications, such as severe vascular dissection, thrombosis, or spasm. The animals were then allowed to recover to a stable state.

### TTE Analysis

Two-dimensional TTE was performed at baseline, 1 h and 1 month post-AMI in all pigs under anesthesia with isoflurane (1.5–2%). Transthoracic images were acquired from a right parasternal approach using a Vivid E9 ultrasound system (GE Vingmed Ultrasound AS, Horten, Norway) equipped with an M5S probe according to the recommendations of the American Society of Echocardiography (23). The images were stored in the DICOM format and analyzed offline using EchoPAC (Version 203). The end-diastolic and systolic volumes (EDV, ESV) were measured from the apical four-chamber view. The left ventricular (LV) ejection fraction (EF) was determined using Simpson's method. A single researcher was blinded to the profile of all animals and analyzed the images.

### Analysis of Plasma Biomarkers and Norepinephrine (NE) in the Renal Cortex

The plasma samples were obtained 15 min after insertion of the arterial sheath, when the animal was intubated under no pain and in a steady state regarding anesthesia at baseline, 1 h and 1 month after modeling. An automatic biochemistry analyzer (Chemray, Guangzhou, China) and the corresponding reagents were used for plasma biochemistry tests. The laboratory parameters measured included blood urea nitrogen (BUN), creatinine, Na+, K+, AST, ALT, DBil, and TBil. Commercial enzyme-linked immunosorbent assay (ELISA) kits were used to determine the plasma concentrations of soluble ST2 (sST2, Presage®ST2 Assay, Critical Diagnostics, Boston, MA, USA), NT-proBNP (CEA485Pro, Cloud-clone, Wuhan, China), renin (H216, Nanjing Jiancheng, Nanjing, China), angiotensin II (H185, Nanjing Jiancheng, Nanjing, China), and aldosterone (H188, Nanjing Jiancheng, Nanjing, China) according to the manufacturer's instructions. The renal cortex was harvested, and the NE concentrations were detected using a commercial ELISA Kit (KA3836, Abnova, Taipei, China).

### Histology Analysis

Histological analysis was performed after 1M-FU. Sample from LV non-ischemic area and renal arteries in both groups were collected and embedded in paraffin. Heart tissue sections were stained with Masson's staining for fibrosis. WGA, CD68, vWF were stained for cell size, inflammatory cells, and angiogenesis (all from Abcam Inc., Cambridge, MA, USA) under established protocol and quantified imageJ (National Institutes of Health, MD, USA). The intensity and algorithm were preset and maintained constant for analysis of all sections. Renal arteries were stained with Tyrosine hydroxylase (TH, Abcam Inc., Cambridge, MA, USA).

### Western Blotting and Quantitative Real-Time Polymerase Chain Reaction

Samples from the non-ischemic area of the LV in both groups were collected. Protein extraction and western blotting were performed as previously described (24). Antibodies used for this experiment were as follows: GAPDH (1:1,000, Abcam Inc., Cambridge, MA, USA), collagen I (Col I, 1:1,000, Abcam Inc., Cambridge, MA, USA), collagen III (Col III, 1:1,000, Abcam Inc., Cambridge, MA, USA), αSMA (1:1,000, Abcam Inc., Cambridge, MA, USA), NF-κb (1:1,000, Abcam Inc., Cambridge, MA, USA), and TGFβ (1:1,000, Abcam Inc., Cambridge, MA, USA).

Total RNA was extracted from the tissues and reverse-transcribed into complementary deoxyribonucleic acid (DP419, TIANGEN, Beijing, China; RR037A, TAKARA, Iwate ken, Japan). Quantitative real-time polymerase chain reaction was performed to detect relative mRNA expression (Bio-Rad, Munich, Germany; RR420L, TAKARA, Iwate ken, Japan). The primer sequences used for the quantitative polymerase chain reaction are listed in Supplementary Table 1.

### Statistical Analysis

Raw data of the groups were given as an input in GraphPad Prism 9.0 (GraphPad Software, San Diego, CA, USA) for statistical analysis. All data are expressed as mean ± standard error of the mean (SEM). Comparisons of the data between groups were performed using Student's *t*-test. Differences were considered statistically significant at *p* < 0.05.

## Results

### Model Establishment and Immediate Evaluation

**Figure 1:**
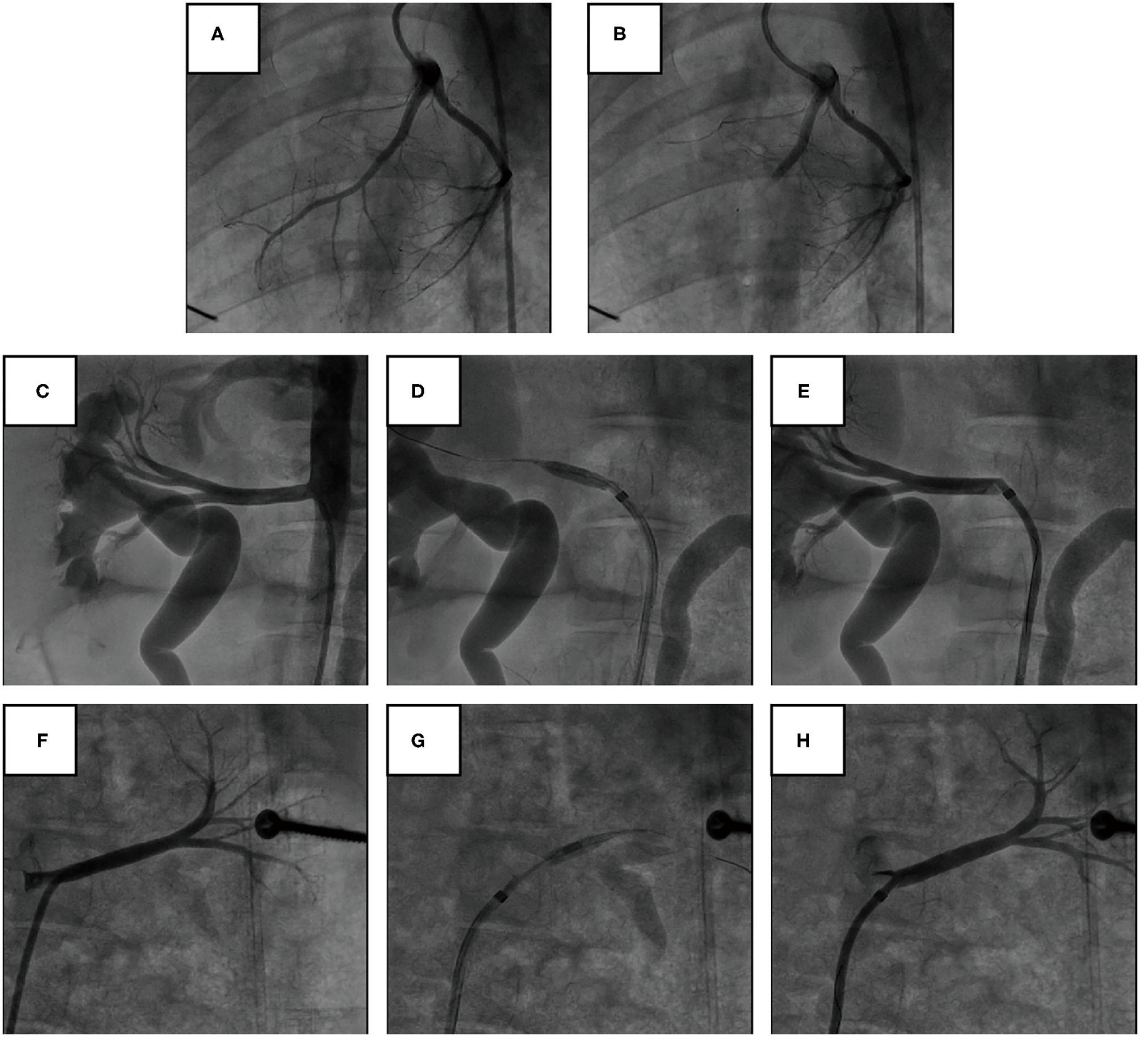
Angiography of coronary and renal arteries in pig models. **(A,B)** Representative angiography of the LAD before and after gelatin sponge embolization. **(C–E)** Representative angiography of the right renal artery before, during, and immediately after cryoablation of RDN. **(F–H)** Representative angiography of the left renal artery before, during, and immediately after cryoablation of RDN.

AMI was successfully induced in all of the 14 pigs (Figures 1A,B). The pigs were randomly assigned to undergo either renal angiography or immediate RDN after 1 h of hemodynamic monitoring. Blood pressure and heart rate were comparable between the two groups at baseline and 1 h post modeling (Table 1). No adverse vascular complications in the renal arteries (e.g., thrombosis, dissection, perforation, and fistulae) occurred immediately after RDN (Figures 1C–H).

**Table 1:** Hemodynamic and biological parameters at baseline, end-procedure and during the 1M-FU.

	**AMI**	**AMI+RDN**	***p***
*N*	6	6	—
Baseline mean arterial pressure, mmHg	75.63 ± 3.96	77.00 ± 6.38	0.65
1 h post-AMI mean arterial pressure, mmHg	69.17 ± 4.66	69.80 ± 6.43	0.87
1M-FU mean arterial pressure, mmHg	74.56 ± 5.75	78.08 ± 6.75	0.35
Baseline heart rate, bpm	76.13 ± 5.69	78.50 ± 5.47	0.48
1 h post-AMI heart rate, bpm	91.50 ± 5.28	87.00 ± 7.90	0.34
1M-FU heart rate, bpm	79.13 ± 5.46	76.33 ± 5.09	0.38
Baseline plasma BUN, mg/dL	8.40 ± 1.77	8.19 ± 2.06	0.85
1M-FU plasma BUN, mg/dL	12.10 ± 1.36	9.58 ± 2.20	0.03
Baseline plasma creatinine, μmol/L	92.94 ± 15.93	89.25 ± 8.48	0.65
1M-FU plasma creatinine, μmol/L	136.87 ± 14.99	115.90 ± 16.11	0.04
Baseline plasma Na+, mmol/L	125.54 ± 5.15	126.62 ± 1.99	0.64
1M-FU plasma Na+, mmol/L	127.01 ± 4.28	126.74 ± 6.93	0.94
Baseline plasma K+, mmol/L	15.32 ± 1.72	14.61 ± 2.75	0.60
1M-FU plasma K+, mmol/L	15.99 ± 1.53	15.67 ± 3.62	0.85
Baseline plasma AST, U/L	28.39 ± 8.72	24.87 ± 5.23	0.44
1M-FU plasma AST, U/L	31.55 ± 10.65	27.92 ± 13.63	0.62
Baseline plasma ALT, U/L	35.31 ± 5.92	39.53 ± 7.84	0.31
1M-FU plasma ALT, U/L	48.04 ± 10.22	48.23 ± 21.45	0.98
Baseline plasma DBil, μmol/L	2.23 ± 0.37	2.23 ± 0.46	1.00
1M-FU plasma DBil, μmol/L	2.74 ± 0.47	2.25 ± 0.57	0.17
Baseline plasma TBil, μmol/L	4.15 ± 0.82	4.17 ± 0.91	0.97
1M-FU plasma TBil, μmol/L	5.64 ± 1.01	4.96 ± 0.97	0.26

Two pigs (one pig in the AMI+sham group and one pig in the AMI+RDN group) died of recurrent ventricular fibrillation in the first 24 h postoperatively during follow-up and were excluded from the statistical analysis. A total of 12 pigs completed the entire 1M-FU process (six pigs each in AMI+sham group and AMI+RDN group).

### Immediate RDN Suppressed SNS and RAAS Overactivity in HF After AMI

**Figure 2:**
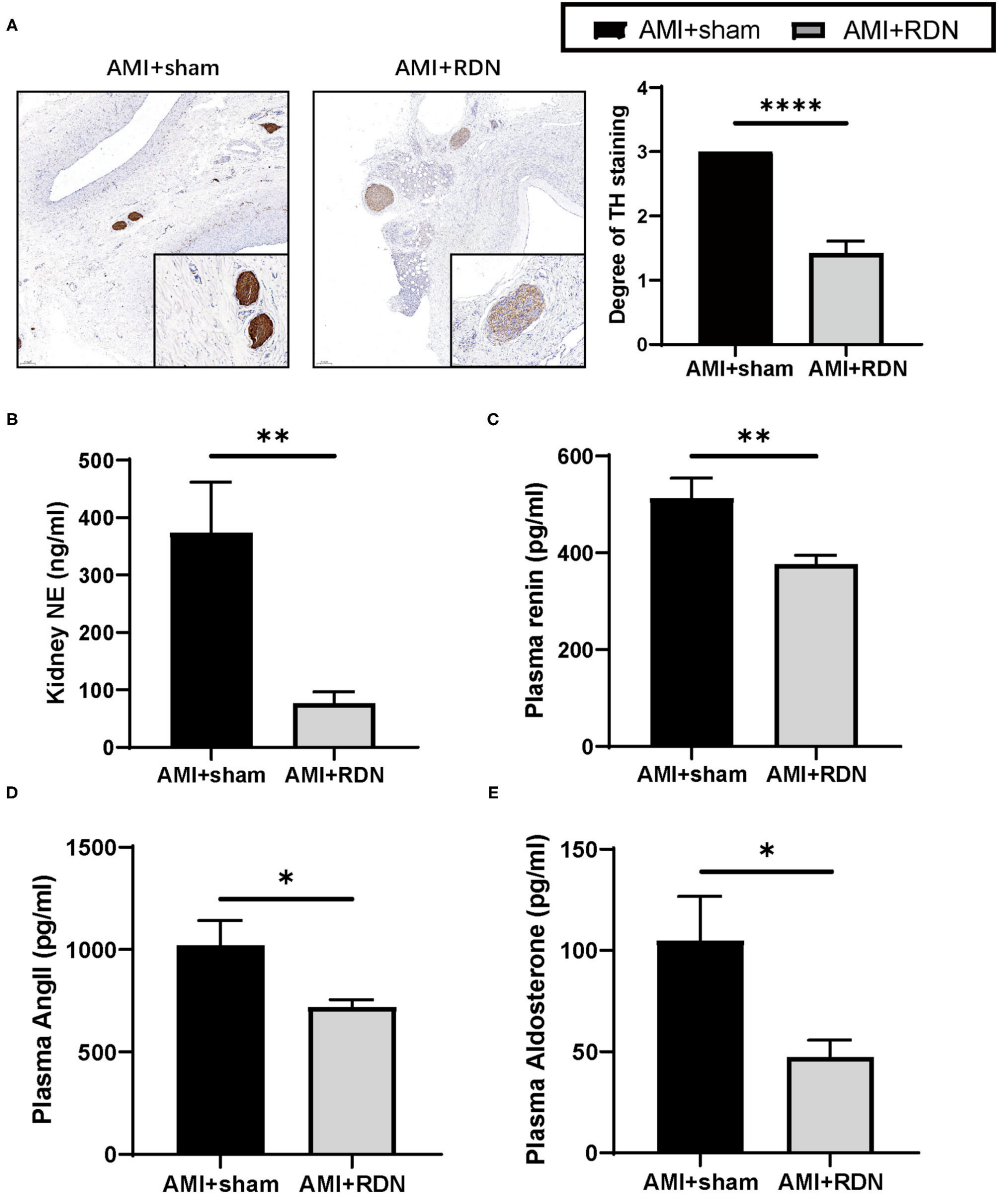
Immediate RDN effectively reduces renal sympathetic activity and plasma RAAS indexes after AMI. **(A)** Representative TH-stained photomicrographs and degree of TH staining of nerves around the renal arteries of pigs from the AMI and AMI+RDN groups on a scale of 0–3: 0, no reaction; 1, patchy/very weak reaction; 2, weak to moderate reaction; 3, strong reaction. The minimum score that represents the most serious functional damage was adopted for each section. **(B)** Quantification of NE concentration per gram of renal cortex of pigs from the AMI and AMI+RDN groups. **(C–E)** Quantification of plasma renin, angiotensin II, and aldosterone levels at 1M-FU of pigs in the AMI and AMI+RDN groups by 1M-FU. Values are expressed as mean ± SEM (*n* = 6). One-way ANOVA was applied to determine the significance of the data. *p < 0.05, **p < 0.01, ****p < 0.0001. TH, tyrosine hydroxylase; NE, norepinephrine; SEM, standard error of the mean.

TH is the rate-limiting enzyme in catecholamine synthesis and is often used to evaluate renal sympathetic nerve activity. At the end of the 1M-FU, renal artery tissues from both groups of pigs were harvested for TH staining and scored for intensity. As shown in Figure 2A, the TH staining intensity and score were significantly lower after immediate RDN in the AMI+RDN group than that in the AMI+sham group. Similar results were also obtained in the measurement of NE, as the NE concentration in the renal cortex of the AMI+RDN group was significantly reduced compared to that in the AMI+sham group (Figure 2B). These results suggest that immediate cryoablation effectively impairs renal sympathetic nerves and their functional activity.

The RAAS system remains continuously hyperactivated during the course of HF in addition to SNS overactivation, and the former plays a decisive role in myocardial remodeling. We found that immediate RDN suppressed the overactivation of RAAS to some extent by analyzing the peripheral blood of the two groups, as evidenced by a significant reduction in plasma renin, angiotensin, and aldosterone levels at the 1M-FU (Figures 2C–E).

Liver and renal function were also examined at the preoperative baseline and at the 1M-FU. The results showed a protective effect of immediate RDN on blood BUN and creatinine in the AMI+RDN group pigs, whereas the plasma potassium and sodium levels and liver function indices (AST, ALT, TBIL, and DBIL) did not differ between the two groups (Table 1).

### Immediate RDN Improved Cardiac Function After AMI

**Figure 3:**
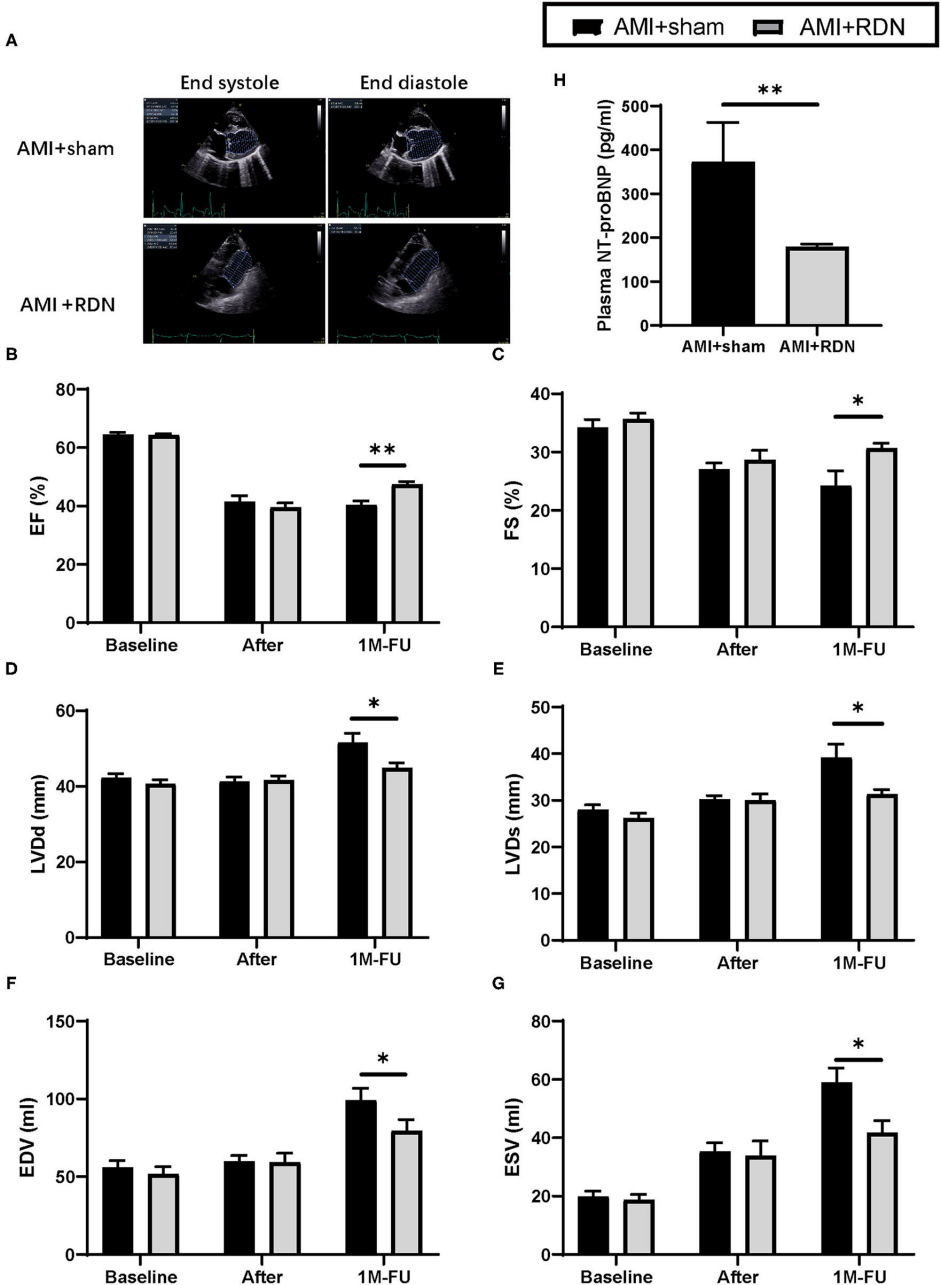
Immediate RDN improves LV function and plasma NT-proBNP level in pigs after AMI. **(A)** Representative echocardiography images of pigs in the AMI+sham and AMI+RDN groups before, after gelatin embolism, and at 1M-FU. **(B–G)** Statistical analysis of EF, FE, LVDd, LVDs, EDV, EVS of pigs in AMI+sham and AMI+RDN groups before, after gelatin embolism, and at 1M-FU. **(H)** Quantification of plasma NT-proBNP level at 1M-FU in pigs in the AMI+sham and AMI+RDN groups. Values are expressed as mean ± SEM (*n* = 6). One-way ANOVA was applied to determine the significance of the data. *p < 0.05, **p < 0.01. EF, ejection fraction; FS, fractional shortening; LVDd, left ventricular end-diastolic dimension; LVDs, left ventricular end-systolic dimension; EDV, end-diastolic volume; ESV, end-systolic volume.

Cardiac function was tested again using TTE at the 1M-FU (Figure 3A). The results indicated that immediate RDN exhibited remarkable improvement in several parameters of cardiac function, as evidenced by higher EF and fraction shortening of the left ventricle in the AMI+RDN group than that in the AMI+sham group (Figures 3B,C). Additionally, immediate RDN also significantly improved LV end-diastolic dimensions, LV end-systolic dimensions, end-systolic volume, and end-diastolic volume (Figures 3D–G).

We then examined cardiac function parameters in the circulation and found that NT-proBNP in the AMI+RDN group was significantly lower than that in the AMI+sham group, further suggesting a protective effect of immediate RDN on cardiac function (Figure 3H).

### Immediate RDN Reduced Myocardial Remodeling in LV After AMI

**Figure 4:**
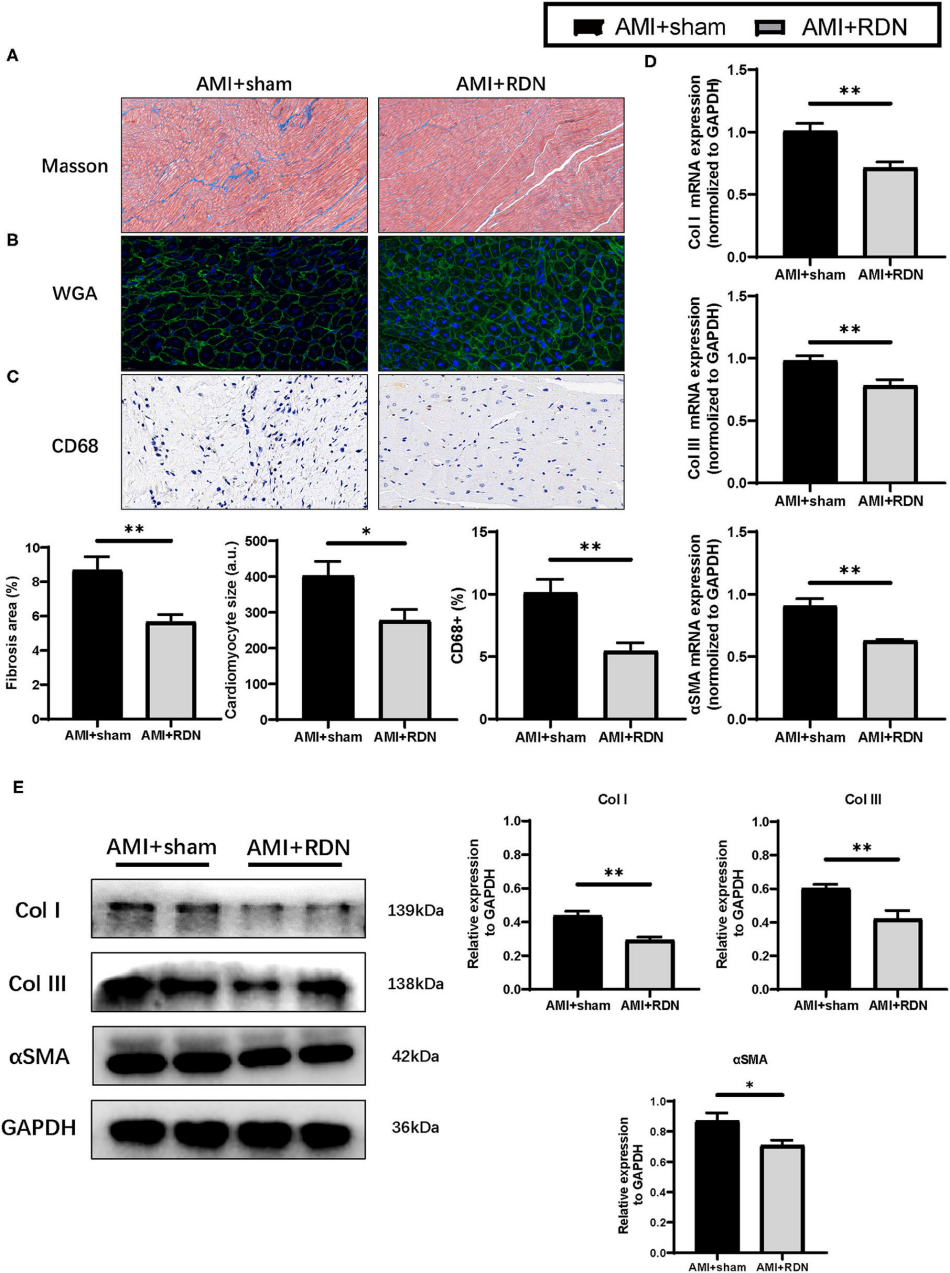
Immediate RDN suppression LV remodeling after AMI. **(A–C)** Representative photomicrographs of Masson trichrome, WGA and CD68 staining and quantitative data of remote area in the extracted heart of pigs in AMI+sham and AMI+RDN groups by 1M-FU. **(D)** Relative mRNA expression of Col I, Col III, and αSMA in remote areas of pigs in AMI+sham and AMI+RDN groups at 1M-FU. **(E)** Representative images and relative protein expression of Col I, Col III, and αSMA in remote areas of pigs in AMI+sham and AMI+RDN groups by 1M-FU Western Blotting analyses. Values are expressed as mean ± SEM (*n* = 6). One-way ANOVA was applied to determine the significance of the data. *p < 0.05, **p < 0.01. a.u., astronomical unit.

Myocardial remodeling is the primary pathological basis of cardiac dysfunction. The results obtained from our study prompted us to explore the influence of immediate RDN on myocardial remodeling. As shown in Figure 4A, the collagen density was dramatically suppressed after immediate RDN at the 1M-FU with the progressive formation of fibrosis in the non-ischemic area. Moreover, cardiomyocytes in AMI+RDN group exhibited smaller sized compared to AMI+sham group (Figure 4B). We also assessed CD68 staining and found out that inflammatory infiltration during HF were inhibited after immediate RDN (Figure 4C). However, vWF staining showed no difference in angiogenesis between the two groups (Supplementary Figure 1).

Similar to the results obtained for the pathological analysis, the elevated mRNA and protein expression of fibrosis-related proteins such as Col I, Col III, and αSMA in remote area of the myocardial tissue after AMI were mitigated to some extent by immediate RDN (Figures 4D,E). These findings implicated a protective effect of immediate RDN in myocardial remodeling.

### IL-33/ST2 Signaling Pathway May Be Involved in the Cardioprotective Effect of Immediate RDN

**Figure 5:**
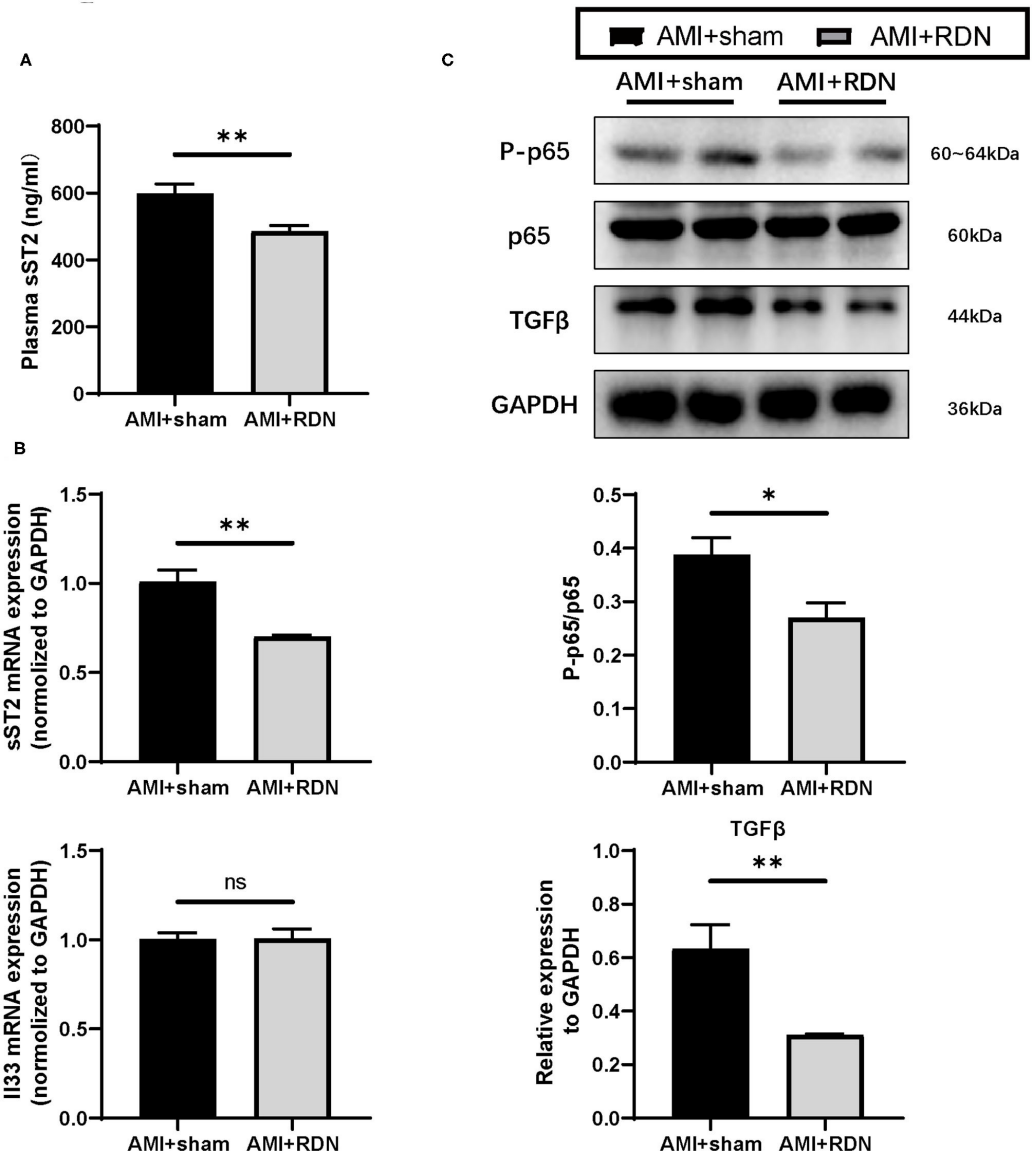
IL-33/ST2 and downstream signaling after immediate RDN. **(A)** Quantification of the plasma sST2 level at 1M-FU of pigs in the AMI and AMI+RDN groups by 1M-FU. **(B)** Relative mRNA expression of sST2 and IL-33 in remote area of pigs in the AMI and AMI+RDN groups at 1M-FU. **(C)** Representative images and NF-κB activation (ratio of P-p65 to total p65) and relative protein expressions of TGFβ in remote area of pigs in AMI and AMI+RDN groups by 1M-FU Western Blotting analyses. Values are expressed as mean ± SEM (*n* = 6). One-way ANOVA was applied for determining the significance of data. *p < 0.05, **p < 0.01. sST2, soluble ST2.

A protective role is played by sST2 in the circulation in myocardial remodeling, and it is now regarded as a prognostic biomarker for HF. Therefore, we wanted to determine whether the protective effects of immediate RDN on LV remodeling was through its effect on sST2. As expected, plasma sST2 levels were significantly lower in the AMI+RDN group than those in the AMI+sham group (Figure 5A). Additionally, sST2 expression in the non-ischemic areas of the LV was also reduced, but the expression level of its cardioprotective ligand IL-33 remained stable (Figure 5B).

NF-κB is important for inflammation and immunity in AMI and has also been reported to be activated by competitive binding of sST2 to IL-33, which promotes the synthesis of TGFβ. We verified downstream NF-κB activation and TGF-β expression to further validate the role of sST2 in the cardioprotective effects of immediate RDN and found significantly suppressed expression of these indicators in the AMI+RDN group (Figure 5C and Supplementary Figure 2). These results implied a protected activation of IL-33/ST2 signaling and the role of decreased sST2 in the anti-remodeling effects of immediate RDN.

## Discussion

In this study, we successfully established a porcine model of AMI and post-AMI RDN. Decreased cardiac function, determined by TTE, was observed postoperatively in pigs of both the AMI+sham and AMI+RDN groups. However, immediate RDN after AMI, which suppressed the SNS and RAAS hyperactivation, exhibited a protective effect on cardiac function and an overall improvement in plasma HF markers after 1 month. The cardioprotective effects of RDN were mainly a result of improved LV remodeling, marked by a reduction in myocardial fibrosis, decreased cardiomyocytes hypertrophy and less inflammatory cell infiltration. Further analysis revealed attenuated expression of sST2 by immediate RDN, together with the involvement of IL-33/ST2 and downstream signaling in the reduction of myocardial remodeling.

In this study, the deterioration in cardiac function after AMI was significantly restrained by immediate RDN with improved TTE parameters and lower plasma NT-proBNP levels at the 1M-FU, suggesting a reduced need for compensatory mechanism. From a pathological perspective, myocardial remodeling persists in the course of HF, manifested mainly by myocardial fibrosis and hypertrophy (2, 20). Our study confirmed for the first time that immediate RDN after AMI significantly repressed fibrosis and cardiomyocytes hypertrophy in the non-ischemic myocardium. Previous studies have suggested that RDN has an antifibrotic role in many other pathological processes, such as delayed HF, renal fibrosis in chronic kidney dysfunction, and atrial fibrosis in arrhythmia (17, 21–24). The protective effect of RDN on myocardial hypertrophy has also been reported in hypertensive models (25). These all supported, to some extent, the results obtained from our findings at both the histological and molecular levels. Infiltration of inflammatory cells is another an important pathological process in remodeling (3). We also found that there were fewer CD68-positive cells in the LV of the AMI+RDN group than in the AMI+sham group. This is cohesive to our previous findings in an atherosclerotic ApoE^−/−^ mice model where RDN mitigated the mobilization of inflammatory cells to plaques (14). Furthermore, we ensured a consistency of the infarct size to a large extent with the use of a permanent embolization model, eliminating the differences in cardiac function caused by this confounding factor, as it is reported that RDN can protect against cell death caused by reperfusion, thus reducing infarct size (26). Collectively, these results for the first time suggest that immediate RDN after AMI is cardioprotective and this effect is mainly exerted by counteracting myocardial remodeling from the onset of HF.

Additionally, we also reported for the first time that the expression of sST2, as a clinical biomarker, declined dramatically in the AMI+RDN group after immediate RDN compared with that in the AMI+sham group. Recently, sST2 has emerged as a prognostic biomarker for HF, and its peripheral level has been reported to reflect the degree of myocardial remodeling (27–29). sST2 is responsible for fibroblast activation, cardiomyocytes hypertrophy, and inflammatory responses in the progression of HF (30, 31). As a decoy receptor, it competitively weakens the binding of IL-33 to ST2L, which inhibits the activation of NF-κb, exerting a direct anti-inflammatory effect on one hand and suppressing the activation of TGFβ and its downstream profibrotic and pro-hypertrophic pathway on the other hand (32, 33). Likewise, the improvement in these phenotypes was also detected as the cardioprotective effect of immediate RDN. In our study, we observed reduced levels of sST2 after AMI by immediate RDN, together with changes in IL-33-related remodeling pathways, including reduced activation of NF-κb and expression of TGFβ. The inhibitory effect of RDN on NF-κb has been demonstrated previously, which further supports our findings that RDN exerts anti-remodeling effects by the reduction of sST2 and the subsequent modulation of the IL-33/ST2 pathway (34, 35).

With the use of a large animal model, this study may provide insights into the clinical application of RDN as it has several advantages. Inconsistence in results happened between large and small animal models (36). Swine models are closer to humans not only morphologically but also physiologically and are more suggestive in practice. The modeling of AMI in swine is also more similar to the pathological formation of AMI in human. Besides, to perform RDN, small animal are treated through open abdominal approaches, and there may be confounding of results due to traumatic strikes. Large animals, on the other hand, are underwent the same interventional approach as humans, and therefore can better simulate clinical treatment. Previously, improvement in exercise endurance and other surrogate endpoints have shown that RDN is protective in both HFrEF and HFpEF patients. In addition to the commonly used imaging or serological indicators in clinical application, our study also confirmed from a pathological perspective that immediate RDN can exert cardioprotective effects by directly restricting myocardial fibrosis and hypertrophy in non-ischemic areas and limiting inflammatory cell infiltration. More importantly, immediate RDN exhibited a significant protective effect on cardiac function while ensuring hemodynamic stability. Although RDN is antihypertensive, and the kidney is one of the most densely distributed sites of sympathetic nerves, RDN does not fully restore their excitability to normal levels, namely not from “too much” to “too little” (37). Moreover, in most of the previous studies, RDN was performed in the middle to late stages of HF, which may lead to a delay in the intervention that RDN would not display a full protective effect early in myocardial remodeling (38–40). From a clinical point of view, our study reaffirms the cardioprotective effects and clinical feasibility of immediate RDN. It suggested AMI as another potential indication for RDN and readmissions for interventions may not be necessary for AMI patients after their HF has progressed.

This study had several limitations. First, the follow-up period was relatively short. We found that immediate RDN exhibited inhibitory effects on myocardial remodeling of non-ischemic myocardial tissues in the first month after AMI, while these pathological progressions in HF last over years. Considering that nerve regeneration after RDN is barely visible and its antihypertensive effect persists for at least 3 years after operation, we presume to a certain extent that immediate RDN may also exert a sustained cardioprotective effect till the middle- and late-stage HF (41, 42). Second, in this study we discovered for the first time that RDN protected the failing heart via the modulation of IL-33/ST2 signaling, yet the detailed mechanism is still unclear. Existing studies have found that changes in IL-33/ST2 pathway are associated with the RAAS system (43, 44). Since RAAS is regulated by RDN, we speculate that RDN may exert a cardioprotective effect on IL-33/ST2 pathway through RAAS and further detailed mechanisms are under exploration. Third, although we confirmed the cardioprotective effects of immediate RDN after AMI, a pre-existing RDN operation is not protective against AMI. The optimal time window for RDN intervention, such as revascularization immediately after RDN, in ischemic HF remains inconclusive. Therefore, relevant large animal experiments are underway to overcome this gap in knowledge.

## Conclusions

Our study showed that immediate RDN after AMI inhibits early SNS and RAAS activation. It exhibited an improvement in cardiac function mainly by suppressing myocardial remodeling. For the first time, we found that these cardioprotective effects of immediate RDN was associated with a reduction in sST2 as well as the involvement of IL-33/ST2 and downstream signaling. This may provide another viable application of RDN beyond hypertension in emergency PCI.

## Data Availability Statement

The original contributions presented in the study are included in the article/Supplementary Material, further inquiries can be directed to either one of or both of our corresponding authors.

## Ethics Statement

The animal study was reviewed and approved by Animal Care and Use Committee of Zhongshan Hospital, Fudan University.

## Author Contributions

JG supervised the study. LS supervised and critically revised the manuscript. HC performed the statistical analyses and drafted the manuscript. HC, RW, JY, ZG, FX, TZ, ZP, CL, and QL performed the experiments. All authors contributed to the manuscript and approved the submitted version.

## Funding

This study was funded by Chinese Academy of Medical Sciences (2019-I2M-5-060 and 2020-JKCS-0154020) and Shanghai Shenkang Hospital Development Center (SHDC2020CR3023B100).

## Conflict of Interest

The authors declare that the research was conducted in the absence of any commercial or financial relationships that could be construed as a potential conflict of interest.

## Publisher's Note

All claims expressed in this article are solely those of the authors and do not necessarily represent those of their affiliated organizations, or those of the publisher, the editors and the reviewers. Any product that may be evaluated in this article, or claim that may be made by its manufacturer, is not guaranteed or endorsed by the publisher.

## Abbreviations

RDN: renal denervation
AMI: acute myocardial infarction
RAAS: renin-angiotensin-aldosterone system activity
TH: tyrosine hydroxylase
PCI: percutaneous coronary intervention
HF: heart failure
LAD: left anterior descending
LV: left ventricular
BUN: blood urea nitrogen
EF: ejection fraction.
