# Supplementary material for: Immediate Renal Denervation After Acute Myocardial Infarction Mitigates the Progression of Heart Failure via the Modulation of IL-33/ST2 Signaling
Source: Front Cardiovasc Med. 2021 Oct 1;8:746934. doi: 10.3389/fcvm.2021.746934 (PMC8517399; doi:10.3389/fcvm.2021.746934)
Supplement: Supplementary file 1 [file Table_1.doc]

Supplementary Material

# Supplementary Table 1

**Supplemental Table 1.** Primers used for quantitative RT-PCR analysis

| **Swine mRNA** | **Forward primer (5´→3´)** | **Reverse primer (5´→3´)** | |
| --- | --- | --- | --- |
| **Housekeeping gene** |  | |  |
| GAPDH | AATTCAACGGCACAGTCAAG | ATGGTGGT-GAAGACACCAGT | |
|  |  |  | |
| **IL-33/ST2 signaling related genes** |  |  | |
| IL-33 | GCTGCATGCCAACAGTAAGG | GATCCGCAGCTTTCTGTCAC | |
| sST2 | AATGTCCCCGACAAGACCAC | ACGATGTGGCTTCTTCCCTG | |
|  |  |  | |
| **Fibrosis-related genes** |  |  | |
| Collagen I | AATCATGCCCTACTGGTGGC | CTTTTCCGGCAGGACCAGAT | |
| Collagen III | CTGGCCTCCCTGGAATGAAG | CACCCTTAGCACCAACAGCA | |
| αSMA | CCAGAGCAATCAGGGACC | AGTTGGTGATGATGCCGTGT | |
| TGFβ | AAGCGCATCGAGGCCATTC | TCCGACGTGTTGAACAGCATA | |
|  |  |  | |
|  |  |  | |
