# Supplementary figures and images for: Immediate Renal Denervation After Acute Myocardial Infarction Mitigates the Progression of Heart Failure via the Modulation of IL-33/ST2 Signaling
Source: Front Cardiovasc Med. 2021 Oct 1;8:746934. doi: 10.3389/fcvm.2021.746934 (PMC8517399; doi:10.3389/fcvm.2021.746934)

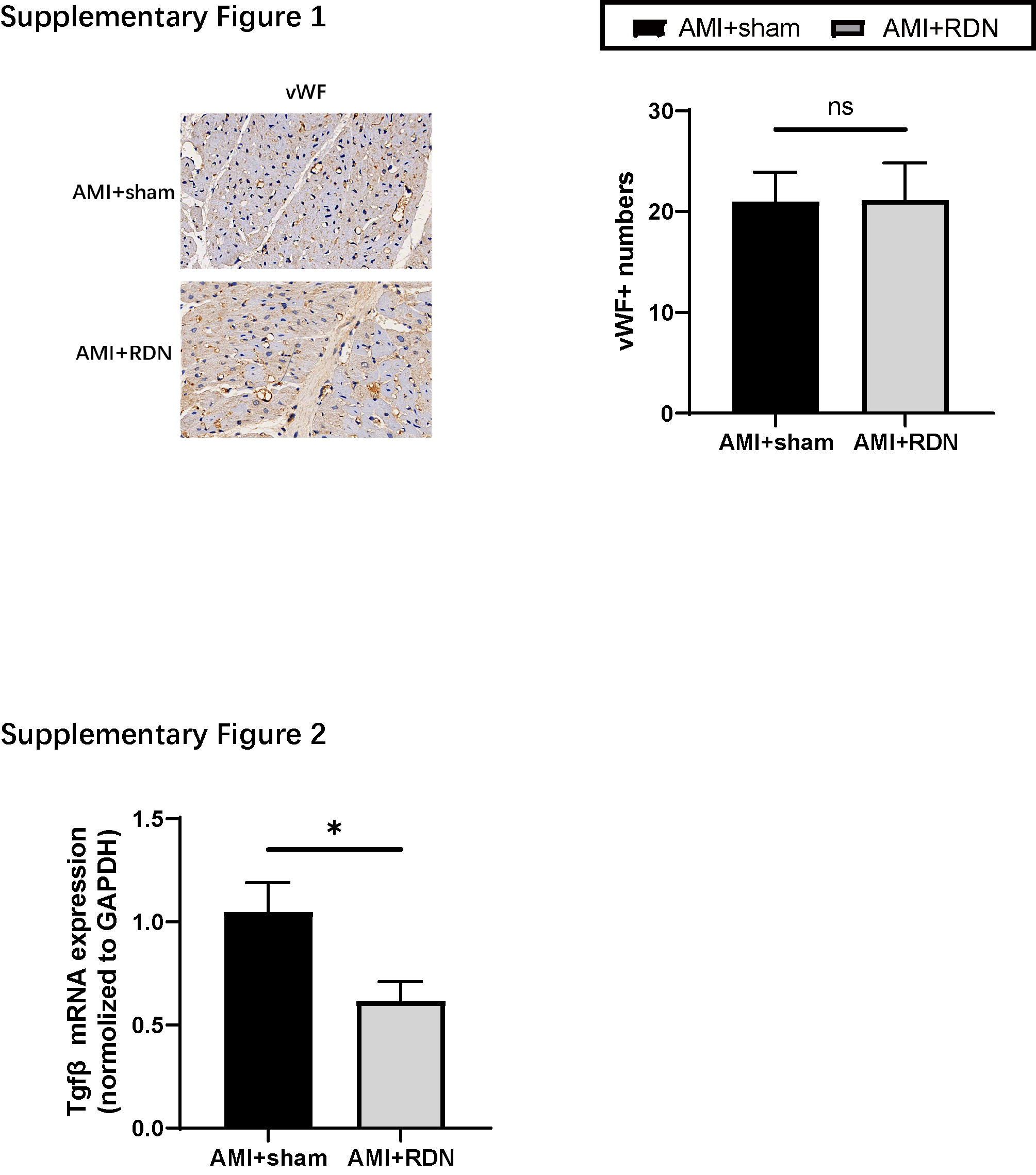

Supplement: Supplementary file 2 [file Image_1.TIF]
